# Safeguarding indigenous learners in schools: a qualitative study of teacher perspectives on child protection policy in Southern Philippines

**DOI:** 10.3389/fpubh.2025.1731249

**Published:** 2026-01-27

**Authors:** Al-Sabrie Y. Sahijuan, Nilo Jayoma Castulo, Nadzmin Jalani Dugasan, Pattra Sakib Ugis, Almira Amilbangsa Hadji, Shernaiza Hajad, Solita Utoh-Asim Jemsy, Russel B. Reyes, Zeda Sangkula, Kharsum Mohammad, Radzmalyn Abdurahman Akmad, Radzmal Soriano Abbani, Alsadjaik Ytang Sabdul, Ajilma Majid Tambihasan, Darwisa K. Sali-Ondo

**Affiliations:** 1 Department of Educational Leadership and Professional Services, College of Education, Mindanao State University - Tawi-Tawi College of Technology and Oceanography, Bongao, Tawi-Tawi, Philippines; 2 Department of Political Science, College of Arts and Social Sciences, Mindanao State University - Tawi-Tawi College of Technology and Oceanography, Bongao, Tawi-Tawi, Philippines; 3 Department of Public Administration, College of Arts and Social Sciences, Mindanao State University - Tawi-Tawi College of Technology and Oceanography, Bongao, Tawi-Tawi, Philippines

**Keywords:** Bangsamoro Autonomous Region in Muslim Mindanao, child protection policy, Filipino teacher, safety, well-being

## Abstract

A significant research gap exists regarding the implementation of child protection policies (CPP) within the unique sociocultural context of the Bangsamoro Autonomous Region in Muslim Mindanao (BARMM) in the Southern Philippines. This qualitative case study aimed to explore public secondary school teachers’ perceptions of the effectiveness of the CPP of the Ministry of Basic, Higher, and Technical Education and the challenges they encounter. Data were collected through key informant interviews and focus group discussions with 10 teachers in Bongao, Tawi-Tawi, and analyzed using inductive thematic analysis. The findings reveal that while teachers perceive the policy as vital for safeguarding students, its effectiveness is hampered by inconsistent implementation, a pronounced lack of continuous training and awareness among teachers and parents, student resistance, and external challenges such as resource limitations and cultural factors. The study’s originality and value lie in its specific regional focus, applying Bronfenbrenner’s Ecological Systems Theory to frame these challenges as interconnected issues across the micro-, meso-, exo-, and macro-levels. It provides context-specific evidence for policymakers and school administrators to design culturally sensitive, multilevel interventions, including robust training, strengthened community collaboration, and addressing systemic barriers, to enhance child protection in similar post-conflict and culturally distinct regions.

## Introduction

The Bangsamoro Autonomous Region in Muslim Mindanao (BARMM) has unique features and a different kind of government setup compared to the rest of the regions within Philippine sovereignty, as stipulated and strengthened through the Bangsamoro Organic Law (RA 11054). In BARMM, basic education is under the management and administration of the Ministry of Basic, Higher, and Technical Education (MBHTE) with seven (7) fundamental principles: inclusivity, equity, right-based, rooted in context, integrated, balanced, and moral governance. The MBHTE-BARMM operates its services under the guidelines and provisions of the Bangsamoro Autonomy Act No. 18, which is known as *An Act Providing for the Establishment, Management, And Support of a Complete and Integrated System of Quality Education in the Bangsamoro* (52). Hence, the MBHTE-BARMM considers the needs and interests of its indigenous peoples, namely, Teduray, Lambangian, Dulangan Manobo, Sama Dilaut (Sama-Bajau), Sama Jama Mapun Sama Daleeyah, Sama Pangutaran, Yakan, and Higaonon, which also include protecting their rights and safeguarding their safety and well-being in school.

Newly qualified teachers often report inconsistent experiences of child protection practices in schools. Many are not adequately informed about their schools’ child protection policies or the designated liaison responsible for these issues (1). Teacher training institutions face challenges in providing adequate training on child protection, which is essential for preparing teachers to interact safely with children and ensure their welfare (2). Similarly, schools must develop child protection policies that reflect their statutory duties and pastoral responsibilities. These policies should address the needs of pupils who have suffered abuse and ensure a safe learning environment (3). In special education schools, administrators prioritize safety through security measures and monitoring. However, the lack of written protocols and limited awareness of legislation can hinder effective implementation (4). Effective child protection requires collaboration between the schools and local authorities. However, there is often a lack of coordination and communication, which can impede the response to child protection concerns (5). Schools play a vital role in identifying and responding to child protection issues. However, their engagement with local authorities and other agencies is crucial for comprehensive protection (6). Furthermore, schools often lack uniform policies and are unfamiliar with state and national provisions for child protection. This can lead to inadequate responses to the disclosure of child sexual abuse and other concerns (5).

There is a notable lack of awareness and understanding of child protection policies and procedures among teachers. Comprehensive training and supportive systems are essential for building resilience among teachers and schools and for promoting a safe environment for children (2, 7). Moreover, Indigenous students face higher odds of out-of-school suspension and involvement in child protective services due to systemic racism, highlighting the need for policies to reduce discipline disparities (8). The use of Information and Communication Technologies (ICTs) in schools exposes learners to risks, such as cyberbullying, inappropriate content, and cyber harassment. Parents, teachers, and learners lack the knowledge and expertise to mitigate these risks, necessitating the development of frameworks to safeguard them (9). Consequently, adequate child protection requires robust evaluation mechanisms to assess the implementation and impact of the policies. Schools need ongoing mentoring and support to ensure the sustainability of child protection programs (10). Improving community outreach and awareness of child protection regulations is crucial. Engaging parents and community organizations in the process can enhance the effectiveness of child protection measures (11, 12). Furthermore, implementing positive schooling approaches that emphasize inclusiveness, strength-based education, and fostering well-being can create healthier and safer school environments. Collaboration among school administrators, counselors, teachers, and parents is essential (12). In addition, the Philippine Department of Education issued Department Order Number 40, series of 2012 (DepEd Child Protection Policy), which has been the context in the Bangsamoro Autonomous Region in Muslim Mindanao through the Ministry of Basic, Higher and Technical Education (MBHTE) Regional Memorandum Number 683, series of 2023 (MBHTE-BARMM Child Protection Policy). Thus, this study explores teachers’ perceptions of child protection policies in public secondary schools in Bongao, Tawi-Tawi. This study takes place in a region of Mindanao with unique educational policies in the Philippines. This study sought to answer the following research question:

1. How do teachers of public secondary schools in Bongao perceive the effectiveness of the Ministry of Basic, Higher, and Technical Education’s child protection policy in ensuring students’ safety and well-being?
2. What are the challenges encountered by teachers of public secondary schools in Bongao in the implementation of the Ministry of Basic, Higher and Technical Education child protection policy?

## Literature review

### Effectiveness of the child protection in ensuring safety and well-being

Public awareness campaigns and professional development programs should be expanded to destigmatize child protection issues and sensitize professionals and society to the importance of early intervention and support. Moreover, child protection training should be mandatory for all professionals working with children, including health care providers, teachers, and social workers. This training should cover the recognition, reporting, and management of child abuse and neglect (13, 14). Likewise, Programs such as Incredible Years, implemented in child protection services, have shown positive impacts on parenting practices and parents’ perceptions of their child’s behavior, although challenges remain in their implementation (15). In Oman, while teachers and caregivers have positive attitudes toward child rights, their knowledge of available child protection services is limited. Participation in awareness activities has been positively associated with the reporting of suspected abuse cases, indicating the need for expanded training programs (16). In addition, collaboration between the University of Iowa and Turkish professionals has led to a significant increase in the number of multidisciplinary teams (MDTs) and improved professional attitudes toward child abuse and neglect. This underscores the importance of culturally competent training programs and policymakers’ involvement of policymakers (17). Consequently, despite increasing awareness, there are significant deficits in the training of health care professionals. Initiatives such as the Medical Child Protection Helpline and online courses in Germany have significantly improved knowledge and competencies among healthcare professionals, highlighting the need for flexible and accessible training options (18, 19). Similarly, in the Philippine educational context, while the Department of Education (DepEd) mandates all teachers to adhere to child protection and welfare standards (see Table 1), the critical need for structured, ongoing training to translate this mandate into effective practice remains a pressing concern.

**Table 1:** Summary of related DepEd memos and policies on Child Protection Policy.

Year	DepEd Memo/No. or Law	Title	Key insights
2012	DepEd Order No. 40, s. 2012	*DepEd Child Protection Policy*	Establishes the official child protection policy that provides guidelines on preventing and addressing child abuse, violence, exploitation, discrimination, bullying, and other forms of abuse in schools. It mandates the establishment of Child Protection Committees (CPCs) in schools and promotes a safe and nurturing environment.
2013	DepEd Order No. 55, s. 2013	*Implementing Rules and Regulations (IRR) of RA 10627 (Anti-Bullying Act of 2013)*	Provides the rules schools must follow to comply with the Anti-Bullying Act. This IRR strengthens the child protection policy’s bullying provisions, requiring schools to adopt and submit anti-bullying/child protection policies and interventions.
2015	DepEd Order No. 18, s. 2015	*Guidelines and Procedures on the Management of Children-At-Risk (CAR) and Children in Conflict with the Law (CICL)*	Offers procedures for dealing with children who are at risk or in conflict with the law, expanding protective measures in school settings.
2017	DepEd Order No. 57, s. 2017	*Policy on the Protection of Children in Armed Conflict*	Defines the department’s role in ensuring that children affected by armed conflict receive protection, continuing the child protection framework beyond typical school issues.
2021	DepEd Order No. 3, s. 2021	*Creation of the Child Protection Unit and Child Rights in Education Desk*	Establishes a dedicated unit and desk within DepEd to coordinate child protection initiatives, integrate child rights in education, and support policy implementation.
2024–2025	DepEd Memo (e.g., OUOPS-2024-05-07998)	*Supplemental Guidelines for the Implementation of DepEd Order No. 40, s. 2012 (CPP)*	Provides additional operational guidance to ensure implementing the Child Protection Policy is effective, clarifies roles, strengthens reporting and response protocols, and updates prohibited acts (e.g., child marriage, OSAEC).

DepEd means Department of Education. Sources. (DepEd Memo (e.g., OUOPS-2024-05-07998), 2025 (46); DepEd Order No. 3, 2021 (47); DepEd Order No. 18, 2015 (48); DepEd Order No. 40, 2012 (49); DepEd Order No. 55, 2013 (50); DepEd Order No. 57, 2017) (51).

### Challenges in implementing child protection in public schools

Limited time resources, staff shortages, and financial constraints complicate the implementation of protection policies (20). Fiscal interventions often suffer from low utilization ratios and infrastructural constraints, which affect their effectiveness (21). Additionally, enhancing policies and strengthening institutional capacity are necessary to bolster legal protection for children (22). In addition, developing collaborative networks between community agencies and organizations is recommended for effective contextual safeguarding (23). Adopting positive schooling, which emphasizes inclusiveness, strength-based education, and collaboration, can create a healthier and safer school environment (24). Nonetheless, limited time resources, staff shortages, and financial constraints complicate the implementation of protection policies (20). Overly bureaucratic responses and a blame culture hinder effective practice reforms in child protection services (25). Cross-sector collaborations face challenges, such as a lack of socio-political support and organizational culture issues, which affect the implementation of programs aimed at reducing racial disparities in child protection (26). Some regions, societal customs, and traditions can hinder the effective implementation of child protection programs. For instance, professionals in Saudi Arabia have reported that customs and traditions are major barriers to implementing child protection training programs (27). A lack of public awareness of the importance of child protection can impede such efforts. In the Banyumas Regency, Indonesia, insufficient public awareness has been identified as a significant obstacle to the implementation of child protection regulations (11). Similarly, the role of community outreach and collaboration among school administrators, teachers, and parents is emphasized in creating a positive school environment (12) Moreover, allocating more funds through regional budgets can help address resource limitations (11).

### Theoretical underpinning

Bronfenbrenner’s Ecological Systems Theory (EST) offers a detailed framework for comprehending human development in relation to various environmental systems that impact an individual (28). This theory has been extensively applied in the study of child development, focusing on how diverse environmental contexts interact to shape growth and behavior (29). The *microsystem* represents the innermost layer, encompassing the immediate environments in which an individual engages directly, such as family, school, and peers, highlighting the reciprocal influences between the individual and their immediate surroundings (30, 31). The *mesosystem* involves connections between these microsystems, such as the interaction between a child’s home and school environments, and how these relationships affect the child’s development (31, 32). The *exosystem* includes broader social settings that do not directly involve the individual but still have an indirect impact, such as parents’ workplaces, community services, and local government policies (31, 32). The *macrosystem* consists of cultural and societal norms, values, and laws that shape other systems, including overarching cultural, economic, and political patterns (31, 32). The *chronosystem* introduces the element of time, considering the changes and continuities in an individual’s environment throughout their life, including transitions and historical events (28, 32). By applying EST, a more comprehensive framework is provided to understand the context of child protection policies in schools, which is crucial for ensuring children’s welfare. This involves a combination of statutory duties, training, inter-agency collaboration, and preventive measures to create a safe and supportive environment for children. However, implementing child protection policies can be challenging because of factors such as a lack of awareness among staff, insufficient training, and the complexity of coordinating with external child protection systems. Research indicates that many newly qualified teachers are not fully informed about their schools’ child protection policies, underscoring the need for improved communication and training (5).

## Methodology

### Research design

A qualitative case study design was utilized to explore secondary school teachers’ perception of the child protection policy anchored on the Philippine Department of Education order and the Bangsamoro Autonomous Region in the Muslim Mindanao Ministry of Basic, Higher, and Technical Memorandum on Child Protection Policy Guidelines and Implementation. The flexibility of the case study method allows for the integration of multiple data sources, thereby providing a holistic and nuanced understanding of real-life experiences (33). By emphasizing the participants lived experiences and narratives, the case study approach legitimizes their voices and highlights their adaptive strategies within unique school contexts (34). Case studies can provide rich, detailed insights that quantitative methods may overlook, making them valuable for exploratory research, theory development, and practical applications (33). Thus, this study determines how teachers perceive the effectiveness of the child protection policy in ensuring student safety and well-being, and the challenges in implementing the child protection policy.

### Research locale and participants

This study was conducted in public secondary schools in Bongao, Tawi-Tawi, Philippines. The Bongao municipality is among the 11 municipalities in the province of Tawi-Tawi, the Philippines. The participants were regular permanent secondary school teachers holding a plantilla item from Teacher-I to Teacher III, and those who had been in the teaching profession for 10 years and more years were identified as study participants. The researchers applied purposive sampling techniques to select the participants such that there were four (4) teachers holding teacher-I items, five (5) holding teacher-II items, and one (1) teacher-III item, such that seven (7) teachers had been in the service for 10 to 19 years, and three (3) had been in the service for 20 to 25 years (Table 2). Having more than 9 years of experience offers a deeper understanding of the law and valuable teaching experiences within a diverse community, such as the Bangsamoro Autonomous Region in Muslim Mindanao. Five or 50 % of the participants were class-advisers while the rest were subject teachers but held other crucial tasks. Having equal numbers of class-advisers and non-advisers gave a balanced perspective and insights to nurture and understand learners well-being, welfare, needs, and interest. Further, to establish gender sensitivity, responsiveness, and perspective, the selection of the participants was almost equal, such that six female participants and four male participants with an overall 10 participants.

**Table 2:** Demographic profiling.

Pseudonym	Gender	Plantilla Position	Years of Experience in Teaching	Position and Assignment	Number of CPP Training Attended
Teacher A	Female	Teacher-I	13 Years	Subject Teacher School Paper Adviser	1
Teacher B	Male	Teacher-II	20 Years -	Class-Adviser Mathematics Coordinator	1
Teacher C	Female	Teacher-II	13 Years	Subject-Teacher Designated Administrative Officer	1
Teacher D	Female	Teacher-I	25 Years	Class-Adviser Supreme Secondary Learners Adviser Guidance Designate	1
Teacher E	Male	Teacher II	16 Years	Subject-Teacher English Coordinator	1
Teacher F	Female	Teacher III	19 Years	Class-Adviser	None Through reading only
Teacher G	Male	Teacher II	17 Years	Class-Adviser	1
Teacher H	Male	Teacher I	20 Years	Subject Teachers	1
Teacher I	Female	Teacher I	10 Years	Division Staff Subject-Teacher	2
Teacher J	Female	Teacher II	15 Years	Class-Adviser	1

CPP means Child Protection Policy.

The Key Informant Interview (KII) interview guide with seven participants and a Focus Group Discussion (FGD) interview guide with three participants were developed through a peer review process and submitted to an expert in educational leadership and educational policy for critiquing and validation. The semi-structured interview guide consisted of key questions on the need for awareness and training, impact on classroom management, community engagement, perceived limitation of the policies, effectiveness in safeguarding students, lack of awareness and training, resistance from students, parental involvement and awareness, implementation challenges, and external influence and environment to determine the perceived effectiveness of the child protection policy ensuring students’ safety and well-being, and elaborate changes encountered by the teachers in the implementation of the child protection policy.

### Data collection and analysis

Ethical approval from the subject-based ethics review of the first author’s institution was obtained prior to data collection (*PEM 301-Approved Code No. 2025–01*). The participants provided informed consent and the researchers explained the objectives of the study, procedure, and confidentiality of the data. The participants were interviewed face-to-face for 60 min and recorded with their permission. Data were transcribed and stored on the researchers personal online drive, and only the researchers team could access the files. After the transcription, an inductive thematic approach was used to elicit participants’ relevant views and experiences, aiming to identify themes from the participants’ narratives. First, three researchers performed data cleaning, wherein the cleaned data were thoroughly reviewed by all other researchers and then compared for consistency. Second, another three researchers were assigned coding, and the three researchers performed line-by-line coding of the data manually such that the codes identified and listed by the three researchers were compared and saturated to ensure accuracy and precision. Third, another three researchers performed the coding through the ATLAS.ti web application and compared it with the manual coding, which was performed to confirm the result and gain reliability and validity. Finally, the researchers agreed on consolidating the final enumeration of the codes both from manual and Atlas.ti version 25, from the transcripts, furthermore these codes were grouped by themes such as awareness and training, impact on classroom management, community engagement, perceived limitation of the policies, effectiveness in safeguarding students, lack of awareness and training, resistance from students, parental involvement and awareness, implementation challenges, and external influence and environment. For better interpretation and clearer analysis of the results, the primary researcher identified and listed three quotes for every theme taken from the responses of the participants, resulting in a total of 10 themes as shown herein. Data saturation was reached when no new codes emerged from the transcript. The final output was subjected to linguistic precision using digital language tools such as Grammarly for enhancement and coherence.

## Results

Table 2 summarizes the themes, codes, and frequencies associated with teachers’ perceived effectiveness of the child protection policy in safeguarding student safety and well-being and the challenges encountered by teachers in the implementation of the child protection policy.

The teachers’ perceived effectiveness of the implementation of the child protection policy shows the vitality of awareness and training (35 codes), emphasizing the training of teachers, parents, and students, awareness of the child protection policy (CPP), student background, proper procedure and protocol, and merit and fitness relevance in safeguarding student safety and well-being. The theme of the impact of classroom management (24 codes) conveys how classroom congestion, guidance activities, academic performance, the class-adviser role, and setting classroom rules influence the effective implementation of the child protection policy. With regard to community engagement (22 codes), the importance of communication, Local Government Unit (LGU) support, community contribution, police involvement, and barangay involvement in achieving better school operations to ensure the safety and well-being of the students. On the other hand, the theme perceived the limitations of the policies (19 codes), revealing the effect of teachers’ adjustment, the digital world, immediate response, situation, and fund allocation on the favorable implementation of the child protection policy in school. In terms of theme effectiveness in safeguarding students (15 codes), it shows the significant contribution of monitoring and evaluation, mental health, security forces, supreme secondary learners, advisers, and designate toward ensuring student safety and well-being.

Regarding the challenges encountered by teachers on child protection policy, various themes uncover the challenges in the implementation of child protection policies in public secondary schools. The predominant theme is implementation challenges (28 codes), highlights additional tasks, schools are open to the public, need to adjust, respect, and trust, and boundaries between teachers and students affect the ability and capacity of teachers regarding child protection policies. Next, the theme of lack of awareness and training (27 codes) stressed the pivotal contribution of school administration, gender sensitivity, coordination, consequences of incidence awareness, and technology to improve the implementation of child protection policies in schools. Parental involvement and awareness (20 codes) reinforce awareness of parental consent, family concerns, problems in the house, parent observations, and mastery of child protection policies. However, the theme of students’ resistance (17 codes) shows that the attitude of students, fairness and just treatment, cyber bullying, students’ consideration, and dishonest students become a challenge for teachers in the implementation of child protection policies. Lastly, the themes of external influence and environment (14 codes), such as cultural interference, outsiders’ presence, social media, learning conditions, and economic status also share teachers’ challenges in the implementation of child protection policies in public schools (Table 3).

**Table 3:** Summary of themes.

Category	Themes	Top five codes	Frequency
Teachers perceived effectiveness of the child protection policy in ensuring student safety and well-being.	1. Awareness and Training	Training (teachers, parents & students) Awareness of CPP Student background Proper procedure and Protocol Merit and Fitness	35
	2. Impact on classroom management	Classroom congestion Guidance activity Academic performance Class adviser role Setting classroom rules	24
	3. Community engagement	Communication LGU supports Community contribution Police involvement Barangay involvement	22
	4. Perceive limited understanding of the policies	Teachers need to adjust Digital world Immediate response Situation Funds allocation	19
	5. Effectiveness in safeguarding students	Monitoring and Evaluation Mental health Security forces Teachers concern Personal hygiene	15
Challenges encountered by the teachers on the child protection policy.	1. Lack of awareness and Training	School administration Gender sensitivity Coordination Consequences of incidence Technology	27
	2. Resistance from students	Attitude of students Fairness and justice Cyber bullying Consideration for students Dishonest students	17
	3. Parental involvement and Awareness	Parental consent Family Problem in the house Parents observation Mastery of CPP	20
	4. Implementation challenges	Additional tasks School is open to public Needs to adjust Respect and Trust Boundaries between students	28
	5. Other external influence	Outsiders presence Social media Culture interference Learning condition Economic status	14
		Total	221

The authors created this table.

### How do the teachers perceive the effectiveness of the child protection policy in ensuring student safety and well-being?

#### Awareness and training

Teachers emphasized the need for continuous training and awareness of child protection policies. They believe that, while some teachers may have a basic understanding, ongoing education is crucial for effective implementation. School administrators must take appropriate steps to ensure that teachers have sufficient knowledge and understanding of their duties and functions. Teachers should be aware of the current trends and situation; thus, a school administration should prepare and organize plans and programs to better the services of the teachers, such as school-based training along CPP and other related topics, team-building for strengthening faculty relationships, educational tours, and benchmarking for adaptation of best practices. The teachers expressed their suggestions and recommendations as follows.

*"What I really want to recommend is to have training... more training seminars regarding the Child Protection policy. To be aware not only of training for teachers, training for students, and also training for parents, but also training for the community as a whole."*

*"Yes, more training seminars regarding Child Protection policy."*

*"They should be because not only should the teacher be there as a whole, but the community should also be there."*

#### Impact on classroom management

Teachers feel that child protection policies can aid classroom management by providing guidelines on how to handle various situations, although they also express concerns about the limitations imposed by these policies on traditional disciplinary methods. The school administration supplements teachers’ needs and provides them with necessary technical assistance in terms of classroom management and instructional supervision with respect to safeguarding students’ welfare and well-being. Participants explained the following:

*"Actually, it helps because we will know from the students who have problems, and it will help to build and to give respect and trust to the students, to respect the teacher."*

*"Actually, it doesn't hinder, sir. It's more like it’s good. Because it guides us teachers on how to handle diverse groups of students*.”

*"Yes. Although, sir, there are times when the Child Protection Policy... But it's okay, sir, so that the conflict can be avoided."*

#### Community involvement

There is a strong belief that child protection is community responsibility, and teachers advocate greater involvement from parents and the community in understanding and implementing these policies. The school administration should take into consideration the vital roles and contributions of the community to schools; thus, schools must strengthen relationships with community people and community leaders in order to gain full support for school programs and activities. Some participants said the following.

*"The role of parents is big in protecting their child... they should also be consulted if their child is doing well".*

*"As the saying goes, it takes the whole community to mold a child. It should be the collective cooperation, both from external and internal stakeholders”.*

*"I also agree with the statement of Sir…that there are moments when it is not always in our hands that the security of the learners is being protected."*

#### Perceive limited understanding of the Department of Education (DepEd) policies

Some teachers expressed that, while the policies are beneficial, they can also hinder effective discipline and classroom authority, leading to a lack of respect from students. The school administration has to take intensive action such as formulating school-based guidelines on school policy with respect to national and regional guidelines and strengthening guidance programs through advocacy and orientation. Some participants stated the following.

*"Because teachers today, because of the implemented child protection policy, are afraid. So, that is one of the reasons most of the students today are not obeying their teachers, they are not more respectful to their teachers".*

*"For me, child policy is irrelevant already. Right? Because we have to protect our community without a policy. Right? Everything is formal."*

*"The problem is the implementation. Most of the time, we are the ones who are often the ones who are affected. Most of the time, there are many students who pass through us."*

#### Effectiveness in safeguarding students

Overall, teachers perceive child protection policies as effective in safeguarding students, particularly in raising awareness about their rights and the importance of a safe learning environment; however, the school should not be complacent of what is explicitly observed that school administration should extend its effort even outside school activities or situations that also include activities in the digital world so that a hundred percent or full effectiveness of the policy is ensured. They confirmed that:

"*For me, sir, it is effective because it increases teachers' awareness and be more responsive... at least in this policy, we will know or help the students who have problems"*

*"For me, yes, it can help. It can help to protect our students, especially if we are aware and if we are going to emphasize this to the students and parents."*

*"I think so, because I believe that in every stage in this curriculum, we keep on learning, we keep on monitoring what's good for the betterment of the learners... the MBHTE with the DepEd is really collaborating as well to do some remedy, to be more efficient in handling this kind of problem."*

### What are the challenges encountered by the teachers in the implementation of the child protection policy in public schools?

#### Lack of awareness and training

Many teachers expressed that they were not fully aware of child protection policies and guidelines, which hampered their effective implementation. Continuous training is seen as essential but often lacking. It is highly recommended that the school administration should strengthen its programs on child protection and children rights through a school-based initiatives like conducting in-house training for teachers on CPP and other important topics like Violence Against Women (VAW), Children Rights, and Lecture along Early Marriage with Islamic perspective and holding an activity that promotes harmonious relationship among students, teachers and parents like Children Month Celebration and Family Day. They further stated the following.

*"I think, as a teacher, maybe they know the CPP. And I can't judge whether they know or not. Maybe they already know because they are teachers and they have seminars. But I think I still need continuous training."*

"*What I really want to recommend is to have training. Yes, more training seminars regarding the Child Protection policy. To be aware not only of training for teachers, training for students, and also training for parents, but also training for the community as a whole."*

*"No, they are not aware. So, they are not aware of that one. Especially, even in the policy, they are not aware. For example, they are permitting their children to get married early. Actually, in the policy, they are not allowed to have this early marriage."*

#### Resistance from students

Teachers reported that some students exhibited disrespect and non-compliance, which they attributed to the protective measures in place that may limit traditional disciplinary actions. The school administration should prepare an action plan on how to mitigate problems such as students’ undesirable behavior, and prepare a student code of conduct so that students will still be aware of the consequences of their actions. The teachers expounded the following.

*"Because teachers today, because of the implemented child protection policy, are afraid. So, that is one of the reasons most of the students today are not obeying their teachers; they are not as respectful to their teachers."*

*"For me, when we talk about child protection policy, it depends. Even for parents, there is a lot of domestic violence. There are students who prefer to stay at school and are afraid to go home."*

*"The attitude of the students is very good, obeying the teachers, then following the rules and regulations. Unlike today, sir. Okay. It's very abusive. There's a lot of bullying."*

#### Parental awareness and involvement

There is a significant gap in parental awareness of child protection policies that affects the overall effectiveness of these measures. Many parents are either unaware of or lack an understanding of the policies. Parents are the best partners of the teachers and school administration in nurturing children and supporting children’s needs, so it is highly recommended that parents should be involved, coordinated, and consulted in all school activities because the offshoot of those activities is for the welfare and best interest of the students or of their child/children. They said that:

"*Most of the parents don't know about the Child Protection policy. But maybe if they know about it, maybe they will implement it in their school."*

*"I think most parents are aware. Because when parents and their children go to school, they feel safe. When they go to school, we ask them. We ask them, Who is bothering you?"*

*"In the CPP, there should be an involvement of parents... there should be a representative from the parents' association, not just teachers."*

#### Implementation challenges

Teachers highlight that while policies exist, the actual implementation is inconsistent. Some teachers do not abide by these provisions, which leads to a lack of uniformity in how child protection is enforced. The school division office should regularly monitor the implementation of the CPP at the school level; provide appropriate guidance and technical assistance to school heads, teachers, staff, and other school personnel; and ensure that CPP is implemented well in schools with strong coordination and cooperation from CPP focal persons and committee members. They noted that:

*"Although they already know and are aware of this department order (DO), there are still teachers who are not implementing because sometimes the teacher is the one who starts the fight in the classroom."*

*"Sometimes, the teacher is also the one who is being bullied. There are things like that. For the teacher to be able to implement, he is the one who is violating the policy. He is the one who is showing how to implement".*

#### Other external influences

Teachers note that external factors such as community violence and social media influence complicate the implementation of child-protection policies. These factors can lead to increased bullying and other issues that the policies aim to address. The school administration must establish good partnerships with all its stakeholders, such as the Barangay Local Government Units, Ministry of Social Services, and even with the Women and Children Affairs and Cyber Crime Units of the Philippine National Police, to mitigate problems that occur outside the school and outside school jurisdiction. The participants mentioned the following.

*"There are many children who are not safe with their parents. I can say that a large portion of the children are safe with their teachers. But for the parents, a large percentage of the children are not safe with their parents."*

*"When the fences are down, when the outsiders are free to go along the way inside the campus of the school, we cannot really assure that the students are safe from the outsiders."*

## Discussion

This case study determines how child protection policies help to ensure the safety and well-being of students in public schools. The findings show that there is a need for a series of substantive training and continuous awareness campaigns, along with child protection, to ensure the safety and well-being of students. The predominant theme from teachers, the urgent need for “continuous training and awareness,” signals a significant gap between policy intent and practical competency. While the literature unequivocally states that training equips teachers to identify and respond to abuse (35) and should be mandatory for all child-facing professionals (13, 14), our findings reveal that the current provision is perceived as insufficient and sporadic. This deficit resides primarily within the *exosystem*, in which inadequate resource allocation for ongoing professional development directly hampers efficacy at the classroom (*microsystem*) level. Furthermore, the call for training to extend to parents and the community underscores a crucial mesosystem disconnect: teachers feel they cannot bear the responsibility alone. This aligns with global research advocating expanded public awareness campaigns to destigmatize child protection and foster community-wide vigilance (19, 36). The success of such interventions in Saudi Arabia, as demonstrated by Temsah et al. (37), reinforces the potential value of this approach. However, our data suggest that in BARMM, this is not merely a recommendation, but a prerequisite for effective implementation, as a lack of parental awareness is frequently cited as a key barrier. Thus, the findings compel us to move beyond viewing training as a simple checklist item and toward framing it as a continuous, multi-stakeholder strategy essential for bridging the policy-practice gap.

As reported by teachers, the challenges in implementing CPP extend beyond the classroom walls, fundamentally highlighting a fractured mesosystem in which the crucial links between schools, families, and the community are weak or non-functional. The pronounced gap in parental awareness and involvement emerged not merely as a challenge but also as a critical failure in the ecosystem meant to protect the child. Teachers reported that parents were often unaware of CPP, which directly undermined its effectiveness. This finding is critical, as the literature confirms that active parental engagement is a cornerstone of positive learner outcomes and safety, from improving discipline and academic performance (38) to mitigating online risks (39). When the school’s and home microsystems are misaligned, as in this case, it creates a vacuum in which student resistance can flourish and protection protocols fail. This necessitates a deliberate move from merely informing parents to actively engaging them as partners, a principle shown to yield broader benefits to family well-being (40). Furthermore, the challenges of “outsiders’ presence” and “cultural interference” cultural interference point to the need for robust community collaboration. As evidenced by successful reforms elsewhere, it is essential to move from a school-centric model to a community-owned safeguarding network. This involves building trust, identifying key protective actors within the community (41) and linking CPP to existing local structures to ensure sustainability (42). Addressing implementation challenges requires a systemic strategy that actively repairs and strengthens mesosystemic connections, thereby embedding child protection as a shared responsibility within the broader social fabric of the BARMM region.

Additionally, the findings highlight the crucial roles of stakeholders, including parents, community leaders, and institutional policymakers, in designing programs and services that ensure student safety, enhance teacher performance, and operate efficiently. A study in Nigeria highlighted that inadequate enforcement of laws against child abuse and exploitation poses a significant issue (43). Meanwhile, initiatives such as Uganda’s urban program on livelihood, income enhancement, and socio-civic transformation intervention (UPLIFT) have demonstrated that bolstering community accountability for child protection services can result in notable progress (44). Similarly, active parental involvement in school activities and programs, particularly those related to child protection, is crucial to fostering a positive and supportive environment for students. Consequently, effective support services should address the needs of both the children and their parents. Engaging parents in the decision-making process through participatory methods can improve the success of these services (45).

### Theoretical implications

The study of child protection policies in public schools in the Philippines with respect to regional contexts, such as in the Bangsamoro Autonomous Region in Muslim Mindanao (BARMM), implies a theoretical implication along the safeguarding of students’ safety and well-being is fundamentally rooted in a holistic approach that encompasses not only the physical environment of the school but also the emotional and psychological dimensions of student experiences in school, at home, and in their environment. Table 2 provides the theoretical implications of this theory in relation to the findings of this study. The findings of this study, analyzed through the lens of Bronfenbrenner’s Ecological Systems Theory, reveal that the challenges in implementing CPP are not merely a result of individual teacher resistance, but are symptomatic of dysfunctions and disconnections across the entire educational ecosystem. At the *microsystem* level, teachers are often caught in difficult positions. CPP is designed to foster trust. However, in practice, some teachers feel that it undermines their authority. This is exacerbated by *mesosystem* failures, particularly lack of parental awareness. When the values and rules of the school (one microsystem) are not reinforced at home (another microsystem), students receive conflicting messages, leading to the resistance and behavioral challenges teachers described. Furthermore, *exosystem* creates significant barriers. The call for more training and resources is a direct response to a system that does not adequately equip teachers with this mandate. Overcrowded classrooms and a lack of support staff are not classroom-level problems; they are the result of resource allocation decisions made at higher administrative levels. This aligns with Tariq (20) and Hariyanto et al. (11), who identified financial constraints and weak institutional capacity as key impediments. Ultimately, this occurs within the unique *macrosystem* of the BARMM. The need for “culturally sensitive approaches” underscores the need for a one-size-fits-all national policy that can be carefully adapted to local customs and realities. The reported “culture interference” is not a simple obstacle but a point of negotiation between the national law and local tradition, a classic macrosystemic dynamic (Table 4).

**Table 4:** Mapping findings with Bronfenbrenner’s ecological systems theory.

Ecological level	Key findings from the study	Manifestation of challenges
Macrosystem	Cultural norms, Early marriage, National/Regional policy	Conflict between policy and local tradition; need for cultural sensitivity.
Exosystem	Lack of training, Limited funds, Overcrowded classrooms	Teachers feel unsupported and underequipped by the system.
Mesosystem	Lack of parental awareness, Weak community links, Inconsistent staff implementation	Efforts in school are undermined by lack of synergy with home and community.
Microsystem	Student resistance, Classroom management issues, Teacher-student boundaries	Daily interactions are strained, affecting the core teacher-student relationship.

The authors tabulated this table.

### Implications toward policy and practice

The theoretical implications extend to the development of policies and frameworks that are culturally sensitive and contextually relevant to the unique cultural and social dynamics of their communities. Practical strategies such as regular training for teachers, parents, and students on child protection policies are essential. The following are the recommendations for policy and practice:

***Design more relevant training and awareness campaigns***: There is a strong need for ongoing training and awareness programs for teachers, parents, and students regarding the Child Protection Policy (CPP). Participants emphasized that awareness should not be a one-time event, but rather a continuous process to ensure that all stakeholders are informed and engaged. Suggestions were made to integrate child protection topics into the school curriculum, particularly in subjects like the values education subject, to enhance students’ understanding of their rights and the policies in place to protect them. Further, the researchers suggested CPP-related training, such as understanding child development and psychology, digital safety and online protection, child protection in indigent community and cultural sensitivity, etc.

***Strengthen parental and community involvement***: Parental involvement is crucial for effective implementation of child protection measures. Many participants noted that parents often lack awareness of CPP, which can hinder its effectiveness. Therefore, initiatives to educate parents about these policies are necessary, and collaboration with local government units (LGUs) and community organizations is essential. Participants highlighted that safeguarding learners requires collective effort from all stakeholders including schools, parents, and community members.

***Addressing implementation challenges***: Many educators have pointed out that overcrowded classrooms and insufficient resources (such as security measures) pose significant challenges for the effective implementation of child protection policies. This environment can lead to increased stress among students and teachers. Similarly, there is a gap in the awareness and understanding of CPP between teachers and parents. While some teachers are familiar with these policies, many are not fully aware of their implications or of how to implement them effectively. Similarly, participants noted that societal issues such as domestic violence and economic pressures significantly impact students’ safety and well-being. Furthermore, the need for culturally sensitive approaches to implementing child protection policies has been emphasized, particularly in diverse communities. Understanding the cultural context of students can help educators to manage and discipline them more effectively.

***Refining classroom management***: Teachers emphasized the necessity of establishing clear rules and boundaries within the classroom. This includes communicating the do’s and don’ts to students, which helps to maintain discipline and order. In particular, the implementation of anti-bullying policies is crucial. Teachers noted that discussing the effects of bullying and ensuring that students understand these policies could create safer classroom environments. Moreover, it is important to be present in the classroom to effectively manage student behavior. When teachers are actively supervising, there are fewer incidents of misbehavior. Hence, ensuring that classrooms are equipped with necessary resources can help maintain discipline and provide a conducive learning environment.

### Limitations and future directions

This study provides significant knowledge and understanding of the implementation of educational policies focusing on child protection in public schools; however, it has several limitations. The findings herein were obtained from a small number of teachers as participants within a specific local context, which many may not represent across all regions of the Philippines. Because the study was mainly based on qualitative narratives, the interpretation emerged only on personal views and experiences, rather than broad generalizations. Future studies should include more participants from different localities and with different types of schools, which may include private schools and basic education schools managed by state colleges and universities to completely represent different situations and perspectives. Further, a longitudinal study and a comparative study between public schools and private schools in the region are highly recommended to produce more substantive and significant outputs. Mixed-methods research is recommended that combines personal narratives and measurable data on teachers’ perceptions and teachers’ encountered challenges in child protection policy.

## Conclusion

This study explores teachers’ perceptions of the Philippine Department of Education Child Protection Policy(CPP) with the aim of determining teachers’ implementation in safeguarding the safety and well-being of students and the challenges faced by teachers in implementing and practicing the child protection policy in public schools. The findings show that safeguarding student safety and well-being is paramount in educational settings. It highlights the critical role that teachers, parents, and the community play in creating a secure environment for learners. Continuous training and awareness programs are essential to ensure that all stakeholders understand their responsibilities regarding child-protection policies. By fostering a culture of safety and respect, schools can empower students to feel secure and supported, which is vital for their overall development and academic success. However, the implementation of child protection policies is challenging. Educators often face resource limitations such as overcrowded classrooms and insufficient support systems, which can hinder their ability to effectively enforce these policies. Additionally, there is a noticeable gap in the awareness and understanding of policies among both teachers and parents, leading to inconsistent application and compliance. These challenges underscore the need for comprehensive strategies that address both logistical and educational aspects of child protection. In a deep sense, while commitment to ensuring student safety is evident, the challenges encountered in implementing child protection policies must be addressed to create a truly safe learning environment. The interplay between safeguarding measures and challenges faced in their implementation highlights the complexity of this issue. By recognizing and tackling these challenges, educational institutions can enhance their efforts to protect students, ensuring that safety is not just a policy but also a lived reality for every student in public schools in the Philippines.

## Data Availability

The raw data supporting the conclusions of this article will be made available by the first author, without undue reservation.

## References

[1] Buckley H McGarry K. Child protection in primary schools: a contradiction in terms or a potential opportunity? Ir Educ Stud. (2011) 30:113–28. doi: 10.1080/03323315.2011.535979

[2] Russell K. Teacher training for child protection In: O’Dea J, editor. Curr. Issues and controversies in Sch. And community health, sport and Phys. Educ New York: Nova Science Publishers, Inc.; Scopus (2012). 81–9.

[3] Taylor AS. From survival to reflection: locating child protection in teacher education. In: Taylor A Garner P, editors. At the crossroads: Special educational needs and teacher education. London: Taylor and Francis; Scopus (2018). 61–74.

[4] Hutchinson AJ O’Leary P Squire J Hope K. Child protection in Islamic contexts: identifying cultural and religious appropriate mechanisms and processes using a roundtable methodology. Child Abuse Rev. (2015) 24:395–408. doi: 10.1002/car.2304

[5] Ramakrishna SK Sharma E Jangam KV Rajendra KM. Gender and personal safety sensitization in schools: a qualitative inquiry into response practices to children’s disclosures of sexual abuse. Indian J Psychiatry. (2025) 67:245–51. doi: 10.4103/indianjpsychiatry.indianjpsychiatry_543_24, 40181869 PMC11964166

[6] Baginsky M Driscoll J Manthorpe J Purcell C. Perspectives on safeguarding and child protection in English schools: the new educational landscape explored. Educ Res. (2019) 61:469–81. doi: 10.1080/00131881.2019.1677167

[7] Daiban S Efthymiou E. Building resilience in the education system: safeguarding children in inclusive settings through teacher empowerment and school preparedness. In: Efthymiou E, editor. Inclusive Phygital learn. Approaches and strategies for students with special needs New York City, USA: IGI Global (2023). 73–96.

[8] Cho M Yoon YJ Flanagan SK Haight W. Students’ risks for out-of-school suspensions: indigenous heritage and child protective services involvement. Child Maltreat. (2023) 28:563–75. doi: 10.1177/10775595231176179, 37201552

[9] Moyo A Tsokota T Ruvinga C Chipfumbu Kangara CT. An E-safety framework for secondary schools in Zimbabwe. Technol Knowl Learn. (2022) 27:1133–53. doi: 10.1007/s10758-021-09545-y

[10] Liestyasari SI Karsidi R Rahman A. Challenges of implementing child-friendly school model in Surakarta, Indonesia. Int J Evaluation Res Educ (IJERE). (2023) 12:2130–7. doi: 10.11591/ijere.v12i4.25149

[11] Hariyanto H Meidina AR Azizah M. Decentralization and the fulfilments of children’s rights: challenges and opportunities for local government in Indonesia. Lex Scientia Law Rev. (2024) 8:677–706. doi: 10.15294/lslr.v8i2.14373

[12] Thomas S Alphonsa Jose K Aneesh Kumar P. Child friendly schools: challenges and issues in creating a positive and protective school environment. In: Deb S, editor. Posit. Sch. And child dev.: Int. Perspect. Singapore: Springer: (2018). 233–48.

[13] Bannon MJ Carter YH. Paediatricians and child protection: the need for effective education and training. Arch Dis Child. (2003) 88:560–2. doi: 10.1136/adc.88.7.560, 12818894 PMC1763165

[14] Mansoor T Mansoor N Ahmed M. Safeguarding children and young people-everyone’s responsibility. J Dow Univ Health Sci. (2019) 13:165–70. doi: 10.36570/jduhs.2019.3.703

[15] Letarte MJ Normandeau S Allard J. Effectiveness of a parent training program “incredible years” in a child protection service. Child Abuse Negl. (2010) 34:253–61. doi: 10.1016/j.chiabu.2009.06.003, 20356626

[16] Al Saadoon M Al Hinai Y Alsabri AA Al Qutaiti HNS Rizvi S. Teachers and caregivers knowledge and attitude about child rights and child protection in Muscat, Oman. Sultan Qaboos Univ Med J. (2025) 25:465–71. doi: 10.18295/2075-0528.2859, 40641713 PMC12244244

[17] Agirtan CA Akar T Akbas S Akdur R Aydin C Aytar G . Establishment of interdisciplinary child protection teams in Turkey 2002-2006: identifying the strongest link can make a difference! Child Abuse Negl. (2009) 33:247–55. doi: 10.1016/j.chiabu.2008.12.008, 19328549

[18] Eberhardt A Hoffmann U Fegert JM Berthold O. Bridging knowledge and practice in the prevention of child maltreatment in medicine: an analysis of counselling and training approaches. Front Public Health. (2025) 13:1–11. doi: 10.3389/fpubh.2025.1588110, 40823217 PMC12350255

[19] Maier A Fegert JM Hoffmann U. “An uncomfortable topic”: health professionals’ perspectives on child protection capacities, training offers and the potential need for action in Germany. BMC Health Serv Res. (2022) 22:1–10. doi: 10.1186/s12913-022-07905-7, 35484623 PMC9052563

[20] Tariq S. A critical examination of the “Schutzkonzept” (protection concept): perspectives and challenges from youth welfare office staff. Findings from a quantitative study. Soz Fortschritt. (2025) 74:55–67. doi: 10.3790/sfo.2024.1451801

[21] Chakraborty L Kaur A Yadav J Balamuraly B. Applying OECD policy evaluation criteria to child protection schemes in Odisha, India. Econ Polit Wkly. (2024) 59:58–65. Available online at: https://www.econstor.eu/bitstream/10419/283223/1/wp_1032.pdf.

[22] Setyawan GA Wijaya AF. Legal protection for children in conflict with the law: policy evaluation and reform recommendations. J Hukum. (2025) 41:157–72. doi: 10.26532/jh.v41i1.42331

[23] Wilson N Diaz C Usubillaga J. Implementing the contextual safeguarding approach: a study in one local authority. J Childrens Serv. (2022) 17:221–36. doi: 10.1108/JCS-10-2021-0043

[24] Stickel M Walter T Götzl C Streb J Dudeck M Fegert JM . Prevention of child sexual abuse: prevention programs and safeguarding concepts in the context of sports, musical education, and religious organizations. Prax Kinderpsychol Kinderpsychiatr. (2024) 73:508–30. doi: 10.13109/prkk.2024.73.6.508, 39290112

[25] Stanley T McGee P Lincoln H. A practice framework for assessments at tower hamlets children’s social care: building on the Munro review. Practice. (2012) 24:239–50. doi: 10.1080/09503153.2012.712677

[26] Boatswain-Kyte A Trocmé N Esposito T Fast E. Child protection agencies collaborating with grass-root community organizations: partnership or tokenism? J Public Child Welf. (2022) 16:349–75. doi: 10.1080/15548732.2021.1891184

[27] Jalal E O’Reilly M Bhakta T Vostanis P. Barriers to implementing learning from child protection training in Saudi Arabia. Int Soc Work. (2019) 62:1493–506. doi: 10.1177/0020872819878485

[28] Neal JW Neal ZP. Nested or networked? Future directions for ecological systems theory. Soc Dev. (2013) 22:722–37. doi: 10.1111/sode.12018

[29] Bizzego A Esposito G. Identifying key physical and natural environmental correlates of child development: an exploratory study using machine learning on data from Pakistan. In: Esposito A Faundez-Zanuy M Morabito FC Pasero E, editors. Smart Innov. Syst. Technol, vol. 360. Singapore: Springer (2023). 351–60.

[30] Bettis J Kakkar S Chan CD. Taking access to the community: an ecological systems framework for in-home counseling with older adults. Adultspan J. (2020) 19:54–64. doi: 10.1002/adsp.12087

[31] Epp A. Bronfenbrenner’s ecological systems theory as a sensitization and examination pattern for empirical analyses. Forum Qual Sozialforsch. (2018) 19:1–14. doi: 10.17169/fqs-19.1.2725

[32] Figueredo AJ Brumbach BH Jones DN Sefcek JA Vásquez G. Ecological constraints on mating tactics. In: Geher G Miller G. Mating intelligence: Sex, relationships, and the mind’s reproductive system. Oxfordshire, UK: Taylor and Francis; Scopus (2007). 337–63.

[33] Yin RK. Case study research and applications: Design and methods. Sixth ed. California, USA: Sage (2018).

[34] Merriam SB. Qualitative Case Studies. In: Peterson P Baker E McGaw B. International encyclopedia of education. Third ed. Amsterdam: Elsevier; Scopus (2009). 456–62.

[35] Gaspar JPM Alcoforado JLM dos Ribeiro Santos EJ Pereira DT. The role of caregivers of children and young people at risk in school context. Rev Conhec Online. (2021) 1:112–26. doi: 10.25112/RCO.V1I0.2389

[36] Grau K Mayer L Haun M Lamp N Berthold O Barzel A . Child welfare risks: (not) an issue!?: explorative analyses of awareness, challenges and training needs regarding child protection in primary care. Z Allg Med. (2024) 100:371–9. doi: 10.1007/s44266-024-00284-8

[37] Temsah M-H Aljamaan F Alhaboob A Almosned B Alsebail R Temsah R . Enhancing parental knowledge of childhood and adolescence safety: an interventional educational campaign. Medicine. (2022) 101:E28649. doi: 10.1097/MD.0000000000028649, 35060555 PMC8772645

[38] Lumadi RI. Taming the tide of achievement gap by managing parental role in learner discipline. S Afr J Educ. (2019) 39:1–10. doi: 10.15700/saje.v39ns1a1707

[39] Vanderhoven E Schellens T Valcke M. Decreasing risky behavior on social network sites: the impact of parental involvement in secondary education interventions. J Prim Prev. (2016) 37:247–61. doi: 10.1007/s10935-016-0420-0, 26821548

[40] Wang Z Bai X. Recharging exhausted parents: how and when involvement in children’s education increases working parents’ flourishing at home and engagement at work. PsyCh J. (2024) 13:780–95. doi: 10.1002/pchj.753, 38627217 PMC11444731

[41] Puma JE Farewell C LaRocca D Paulson J Leiferman J. Community-based mental health interventions for families with young children. In: Osofsky JD Fitzgerald HE Keren M Puura K, editors. WAIMH Handb. Of infant and early child. Mental health: Cultural context, prevention, intervention, and treatment, vol. 2. Cham: Springer (2024). 451–70.

[42] Ellermeijer REC Robinson MA Guevara AF O’Hare G Veldhuizen CIS Wessells M . A systematic review of the literature on community-level child protection in low- and middle-income countries. Vulnerable Child Youth Stud. (2023) 18:309–29. doi: 10.1080/13548506.2023.2230889

[43] Osaiyuwu AB. Exploring the challenges of child protection in Nigeria. In: Levy S Okoye UO Tanga PT Ingram R, editors. Routledge Handb. Of African social work education. London: Taylor and Francis; Scopus (2024). 107–17.

[44] Doh D Kamara J Galukande M Renzaho A. Evaluating the impact of a community-based livelihood intervention on child protection: a mixed method approach. Child Fam Soc Work. (2022) 27:217–35. doi: 10.1111/cfs.12874

[45] Devaney C Crosse R. Key learning and final remarks: parenting support and parental participation. In: Devaney C Crosse R. International perspectives on parenting support and parental participation in children and family services. Amsterdam: Taylor and Francis; Scopus (2023). 284.

[46] DepEd Memo. Supplemental guidelines for the implementation of DepEd order no. 40, s. 2012. Pasig City: (CPP) Department of Education (2025).

[47] DepEd Order No. 3. Creation of the child protection unit and child rights in education desk. Pasig City: Department of Education (2021).

[48] DepEd Order No. 18. Guidelines and procedures on the Management of Children-at-Risk (CAR) and children in conflict with the law (CICL). Pasig City: Department of Education (2015).

[49] DepEd Order No. 40. DepEd child protection policy. Pasig City: Department of Education (2012).

[50] DepEd Order No. 55. Implementing rules and regulations (IRR) of RA 10627 (anti-bullying act of 2013). Pasig City: Department of Education (2013).

[51] DepEd Order No. 57. Policy on the protection of children in armed conflict. Pasig City: Department of Education (2017).

[52] Bangsamoro Parliament. Ba-act no. 18 (Bangsamoro Education Code of 2021) – BARMM. (2021). Available online at: https://officialgazette.bangsamoro.gov.ph/2022/02/23/ba-act-no-18/ (Accessed January 09, 2026).

